# El diálogo oculto entre el hueso y los tejidos a través del remodelado óseo

**DOI:** 10.1515/almed-2023-0101

**Published:** 2023-09-22

**Authors:** María Luisa González-Casaus

**Affiliations:** Medicina de Laboratorio, Hospital Universitario La Paz, Madrid, España

**Keywords:** actividad endocrina, esclerostina, osteocalcina, remodelado óseo, Wnt/β-catenina

## Abstract

El hueso es mucho más que un reservorio de calcio y fósforo. Su disposición lacuno-canalicular ofrece una importante vía de intercambio con la circulación y actualmente, el esqueleto se considera un gran órgano endocrino, con acciones que van más allá del control del balance fosfocálcico mediado por el factor fibroblástico 23 (FGF23). Paralelamente al efecto modulador de las adipoquinas sobre el remodelado óseo, diversas proteínas óseas, como la osteocalcina y la esclerostina, ejercen cierta acción contra-reguladora sobre el metabolismo energético, posiblemente en un intento de asegurar los enormes requerimientos energéticos del remodelado. En esta interacción del hueso con otros tejidos, especialmente el adiposo, participa la señalización canónica Wnt/β-catenina y por ello la esclerostina, una proteína osteocítica que inhibe esta señalización, emerge como un potencial biomarcador. Es más, su participación en diversas patologías le posiciona como diana terapéutica, existiendo un anticuerpo anti-esclerostina, recientemente aprobado en nuestro país para el tratamiento de la osteoporosis. Esta revisión aborda el carácter endocrino del hueso, el papel de la osteocalcina y, especialmente, el papel regulador y modulador de la esclerostina sobre remodelado óseo y la homeóstasis energética a través de su interacción con la señalización canónica Wnt/β-catenina, así como su potencial utilidad como biomarcador.

## Introducción

El hueso es un tejido conectivo especializado con importantes funciones. Constituye el soporte estructural que permite la acción mecánica de tejidos blandos, como la contracción muscular o la expansión de los pulmones, ofrece protección a órganos vitales (cerebro, corazón o pulmones), alberga la médula ósea y es el principal reservorio de calcio y fósforo del organismo.

El 99 % del hueso es una matriz extracelular, integrada fundamentalmente por carbonato de hidroxiapatita. El colágeno tipo I es la proteína mayoritaria de la fracción orgánica de esta matriz y el resto minoritario de proteínas son proteoglicanos, proteínas de la matriz: glicoproteínas (sialoproteína ósea, osteopontina, Dmp1, MEPE, etc.) y GLA proteínas (osteocalcina, proteína GLA de la matriz), así como citoquinas (IL1, IL6) y factores de crecimiento (TGFβ, BMPs). Atrapadas en esa matriz, coexisten diferentes células: adipocitos, células de músculo liso vascular, macrófagos y fundamentalmente osteocitos, osteoblastos y osteoclastos, estando estas tres últimas directamente involucradas en la actividad metabólica del hueso o remodelado óseo.

El remodelado es un proceso dinámico caracterizado por fases coordinadas y secuenciales de resorción osteoclástica y formación osteoblastica, inducidas y moduladas por diferentes factores: mecánicos, hormonales y locales. El osteocito juega un papel crucial en el control de esta actividad al comunicarse con los osteoblastos y los osteoclastos, mediante la expresión de proteínas señalizadoras como el FGF23, el ligando del receptor activador del factor nuclear-κB (RANKL), el Dickkopf-1 (DKK1) y la esclerostina. Gracias a esta actividad de remodelado, el hueso puede adaptarse continuamente a las necesidades mecánicas y bioquímicas del organismo; de hecho, se calcula que el esqueleto adulto se renueva cada 10 años [1]. El mantenimiento de esta estructura y funcionalidad exige un gran consumo energético y evidencias recientes sugieren la participación de algunas proteínas óseas, como la osteocalcina [2] o la esclerostina [3] en la regulación de la homeostasis energética para asegurar estos requerimientos. Esta interacción del hueso con el metabolismo energético, junto al reconocimiento previo de la expresión osteocítica del FGF23 en el control del balance del fosforo, supone una revolución en la percepción del hueso, considerándose actualmente el órgano endocrino más grande del organismo.

Esta revisión plantea el carácter endocrino del hueso; las conexiones “hueso-riñón-intestino-paratiroides” mediadas por el FGF23, y “hueso-páncreas” a través de la osteocalcina. Finalmente, en especial, se aborda la esclerostina, una proteína de origen osteocítico que regula la actividad metabólica del hueso y la modula con la homeóstasis energética a través de su interacción con la señalización canónica Wnt/β-catenina, un elemento clave en el desarrollo y la homeostasis de diversos órganos en la edad adulta. Al inhibir esta señalización, la esclerostina está directamente involucrada en la fisiopatología de diversas enfermedades óseas, por lo que emerge como potencial biomarcador y diana terapéutica.

## Vías de señalización que regulan la actividad metabólica del hueso

**Figura 1:**
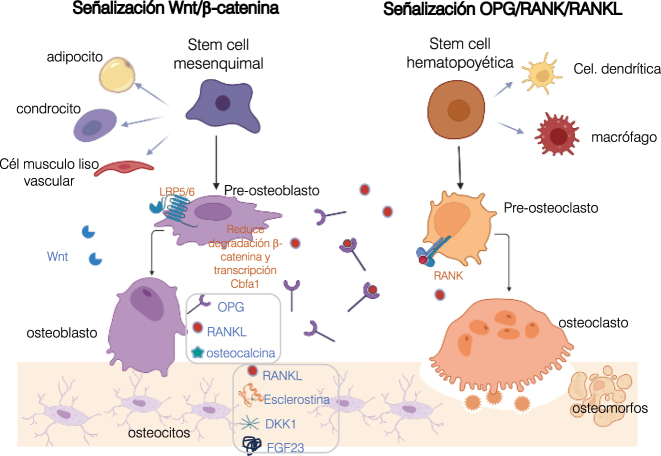
Vías señalizadoras del remodelado óseo. La señalización canónica Wnt/β-catenina es crucial en la diferenciación a estirpe osteoblástica de la célula pluripotencial mesenquimal, común a condrocito, adipocito y célula de músculo liso vascular. La activación del LRP5/6 por los ligandos del Wnt, reduce la fosforilación y degradación de la β-catenina iniciándose la transcripción de genes pro-osteogénicos y la osteoblastogénesis. Los osteocitos, diferenciación final de los osteoblastos, forman una red de comunicación, regulando el remodelado a través de la secreción de proteínas (esclerostina, DKK1, RANKL y FGF23). La esclerostina y el DKK1, inhiben la señalización Wnt al interaccionar con el LRP5/6. Aunque la principal función de los osteoblastos es sintetizar la matriz extracelular, también secretan factores reguladores de la osteoclastogénesis, como el RANKL y la OPG. La señalización del RANK por el RANKL promueve la diferenciación osteoclástica del precursor hematopoyético pluripotencial (común a macrófagos y células dendríticas), la fusión y la activación de osteoclastos e inhibe su fisión a osteomorfos. La OPG antagoniza esta señalización, al actuar como receptor “señuelo” del RANKL y bloquear su unión al receptor RANK. Los osteoclastos también liberan factores que estimulan la formación ósea (BMP6, Wnt10b) *Diseñada con BioRender.com*. LRP5/6, Low density lipoprotein-receptor related protein 5 or 6; DKK1, Dickkopf-1; RANKL, ligando del receptor activador del factor nuclear KB; FGF23, Factor de crecimiento fibroblástico 23; OPG, osteoprotegerina.

Las bases moleculares de esta actividad de remodelado dependen de la interacción de dos vías de señalización: la vía canónica del Wnt/β-catenina que regula la osteoblastogénesis y la vía RANK/RANKL/OPG/(LGR4) que controla la osteoclastogénesis (Figura 1).

### La vía canónica del Wnt/β-catenina

**Figura 2:**
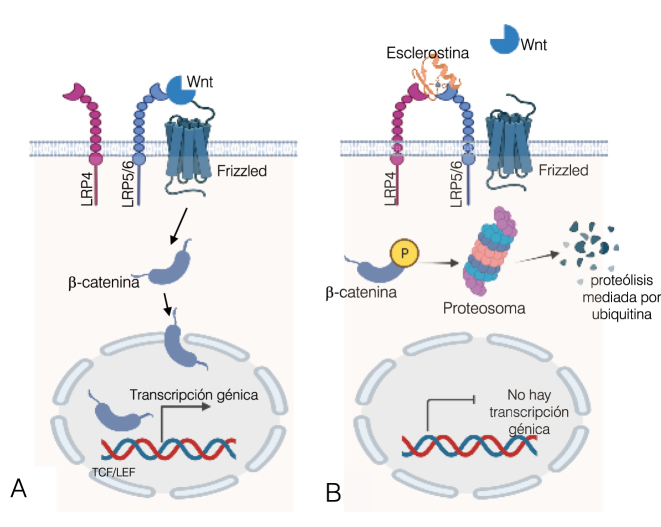
Señalización Wnt/catenina y su inhibición por la esclerostina. (A) Al interaccionar los ligandos Wnt con el receptor LRP5/6 y activar su señalización, se reduce la fosforilación de la β-catenina a permitiendo su translocación al núcleo, donde desplaza represores, activa el complejo de transcripción TCF/LEF e inicia la transcripción de genes pro-osteogénicos (*Cbfa-1*) que estimulan la diferenciacion osteoblástica. (B) La inhibición de esta señalización por la esclerostina se realiza a través del receptor LRP4 que “secuestra” a esta proteína osteocítica para facilitar su interacción con el LRP5/6, e impedir la activación del mismo por los ligandos Wnt. Al inhibir su señalización, la β-catenina se fosforila y se degrada en el proteosoma por ubiquitinación, no pudiéndose realizar la transcripción génica. *Figura diseñada con Bio-render.com*.

El elemento clave de la señalización canónica Wnt es la regulación de la estabilidad proteica de la β-catenina, que actúa como un factor de transcripción. Su activación depende de los ligandos Wnt, unas glicoproteínas secretadas ricas en cisteína, muy conservadas a lo largo de la evolución y capaces de activar al receptor LRP5/6 (low-density lipoprotein-receptor-related protein-5 or -6). En el tejido óseo, la señalización canónica Wnt/β-catenina induce la diferenciación de la célula pluripotencial mesenquimal (precursora común de condrocito, célula de musculo liso vascular y adipocito) a estirpe osteoblástica. La activación del receptor LRP5/6 por los ligandos Wnt reduce la fosforilación de la β-catenina permitiendo su translocación al núcleo, donde desplaza represores, activa el complejo de transcripción TCF/LEF e inicia la transcripción de genes pro-osteogénicos (como el *Cbfa-1*) [4] que estimulan la diferenciación osteoblástica y la formación ósea (Figura 2A). En ausencia de ligandos Wnt, la β-catenina citosólica interacciona con otros componentes del complejo de destrucción (axina 1, APC, GSK-3, CK1), fosforilándose por las dos kinasas de este complejo (GSK-3 y CK1) y degradándose en el proteosoma [5].

La importancia de esta señalización en el desarrollo y en la homeóstasis del tejido óseo adulto implica la necesidad de una fina regulación por inhibidores y activadores [6]. Entre los primeros destacan proteínas que bloquean la señalización al interaccionar con el LRP5/6 (DKK1 y esclerostina) (Figura 2B), proteínas que interaccionan con los ligandos Wnt impidiendo su unión al LRP5/6 (proteínas frizzled secretadas sFRPs 1–5, factor inhibidor del Wnt1 y klotho secretado) o incluso factores inducidos por los Wnt que degradan el LRP5/6 (ZNRF3). Por el contrario, la Norrina y R-spondinas (Rspo) son activadores, que al unirse a los ligandos Wnt promueven su interacción con el LRP5/6. Existen patologías asociadas a alteraciones en la señalización Wnt que desencadenan cuadros de osteoporosis precoz y riesgo de fractura, como las mutaciones con pérdida de función del LRP5 (síndrome de osteoporosis y seudoglioma) o mutaciónes de los Wnt [7, 8].

### Sistema RANK/RANKL/OPG/(LGR4)

Aunque la principal función de los osteoblastos es sintetizar los componentes orgánicos de la matriz, también regulan la osteoclastogénesis mediante la secreción de diversos factores con acciones paracrinas como el RANKL, el factor estimulador de colonias de monocitos (M-CSF), la osteoprotegerina (OPG) o los genes Wnt de la familia 5A (WNT5A) y 16 (WNT16). Entre éstos, destacan el RANKL y la OPG, dos proteínas de la familia TNF, de cuyo equilibrio depende la diferenciación y activación osteoclástica. Los osteoclastos, macrófagos y células dendríticas, proceden de un mismo precursor hematopoyético pluripotencial y su diferenciación a estirpe osteoclástica requiere la activación del receptor activador del factor nuclear-κB (RANK), situado en la superficie celular de los osteoclastos y su precursores, por el RANKL. La interacción RANK/RANKL estimula la diferenciación y fusión osteoclástica, promueve su adherencia al hueso y la activación de osteoclastos preexistentes, inhibiendo simultáneamente su apoptosis [9]. La expresión de RANKL está bajo el control de hormonas (paratirina (PTH), calcitriol, glucocorticoides), citoquinas (IL1, IL6, IL11) y factores de crecimiento (FGF y PDGF) [10]. El incremento neto en el número y actividad osteoclástica, condiciona un aumento de la resorción ósea mediante la secreción de ácido y enzimas líticas (fosfatasa ácida tartrato resistente y catepsina K) que degradan el compartimento extracelular. En este proceso de resorción los osteoclastos liberan asímismo, diversos factores que estimulan la formación ósea (BMP6, WNT10B) o que participan en la respuesta inmune (clastoquinas) [11].

Antagonizando esta señalización, la OPG sintetizada en los osteoblastos y en las células estromales, actua como receptor señuelo uniéndose al RANKL e impidiendo su interación con el RANK [7]. De este modo, ejerce un efecto inhibidor sobre la diferenciación y activación osteoclástica, promoviendo simultáneamente la apoptosis de los osteoclastos, aunque estudios recientes demuestran que, en lugar de este proceso apoptótico, los osteoclastos experimentan procesos de fisión en células hijas más pequeñas y móviles, denominadas osteomorfos, capaces de migrar y fusionarse con otros osteoclastos [12]. La rápida fusion de estos osteomorfos para formar osteoclastos explicaría, en parte, el incremento del riesgo de fractura ocasionado tras la suspensión del Denosumab (anti-RANKL que emula a la OPG). La expresión de OPG está regulada por diversos factores; se incrementa por citoquinas (TNFalfa, IL1, IL18 TGFbeta), proteínas morfogénicas (BMPs) y hormonas esteroideas (17beta estradiol) y disminuye por los glucocorticoides, la PTH y la prostaglandina E2 [11].

Clásicamente, se ha considerado al RANK como el único receptor del RANKL. Actualmente, se reconoce al menos un segundo receptor: el LGR4 (leucin-rich-repeat-containing G-protein-coupled receptor 4), que compite con el RANK, suprimiendo su señalización durante la diferenciación osteoclástica. Al unirse al RANKL, el dominio extracelular soluble del LGR4 regula negativamente la diferenciación osteoclástica y la resorción ósea *in vivo* [13]. Recientes evidencias muestran la participación del LGR4 en el desarrollo de múltiples órganos y en la modulación de alteraciones inmunológicas, metabólicas y en la carcinogénesis [14].

### Papel del osteocito en el control de estas vías señalizadoras

Los osteocitos, las celulas mas diferenciadas de la estirpe osteoblástica, representan el 90–95 % del total de celulas óseas y se disponen formando una estructura reticular atrapada en la matriz mineralizada. Constituyen una red de comunicación que responde ante estímulos mecánicos y hormonales, regulando y coordinando la función osteoblástica y osteoclástica. Por ello, los osteocitos tienen gran importancia en el control del remodelado y la mineralización ósea, actuando como células endocrinas que sintetizan moléculas clave como el RANKL, DKK1 y esclerostina, proteínas señalizadoras que, posiblemente en el futuro, se utilizarán en el manejo clínico de la enfermedad óseo-metabólica, al reflejar los procesos biológicos específicos del hueso y su interacción con otros tejidos.

## El hueso: un gran órgano endocrino

Todas las células presentes en el tejido óseo, incluyendo las stem cell mesenquimales y los adipocitos, pueden sintetizar y secretar una gran variedad de moléculas bioactivas como proteínas, polipéptidos, citoquinas, factores inflamatorios, adipoquinas (leptina, adiponectina) e incluso exosomas para regular el remodelado óseo. Paralelamente, la disposición lacuno-canalicular del hueso ofrece una importante vía de intercambio con la circulación y la presencia de estos factores óseos en sangre sugiere su posible acción extraósea. De hecho, más allá de la función paracrina de estas células, diversas evidencias demuestran que estos factores, al ser liberados a la circulación, actúan en órganos a distancia, confirmándose cada día más el carácter endocrino del esqueleto.

### FGF23: conexión hueso-intestino-riñón-paratiroides

La identificación del FGF23 en los raquitismos hipofosfatémicos familiares [15] revoluciona el conocimiento sobre la homeóstasis mineral. El hueso deja de considerarse un simple órgano diana y emerge como órgano endocrino, conectando directamente el hueso con el intestino, el riñón y la paratiroides, a través de esta proteína osteocítica. El FGF23 es, junto a su correceptor klotho, la principal hormona reguladora del balance del fósforo [16], defendiendo al organismo de la nociva retención crónica de fosfato mediante su acción fosfatúrica y su efecto supresor sobre el calcitriol y la PTH. En la actualidad representa un interesante biomarcador en patologías como la enfermedad renal crónica (ERC), donde el hiperfosfatonismo secundario, aún siendo un mecanismo adaptativo, pasa factura al organismo [17]. Y sobretodo, es una valiosa herramienta diagnóstica en las alteraciones primarias que cursan con exceso de FGF23, como los raquitismos hipofosfatémicos o los tumores inductores de osteomalacia donde, además es diana terapeútica tras el desarrollo de un anticuerpo anti-FGF23 (Burosumab) [18]. Aunque su principal función es el control del fósforo, se han sugerido otras acciones adicionales sobre el metabolismo del hierro, la inflamación y la eritropoyesis, e incluso sobre las funciones cognitivas [19]. A nivel paracrino, también parecen existir conexiones entre el FGF23 y algunas alteraciones del remodelado óseo, como las mutaciones del Wnt1 que ocasionan osteoporosis precoz y fractura prevalente. A pesar de la severa afectacion ósea, los marcadores clásicos de remodelado óseo (MROs) e incluso los inhibidores de la señalización Wnt (DKK1 y esclerostina) no están alterados, siendo el FGF23 circulante el único biomarcador aumentado [20].

### Osteocalcina: conexión hueso-páncreas-metabolismo energético

Poco después del reconocimiento del hueso como organo endocrino involucrado en la homeóstasis mineral, el grupo de Karsenty observa, mediante manipulación genética en ratones, que igual que el tejido óseo se ve afectado por hormonas como la adiponectina, leptina e insulina, existe un mecanismo de feedback en el control del metabolismo energético a través de la osteocalcina [2]. La osteocalcina, una proteína de expresión osteoblástica (es marcador de diferenciación osteoblástica en cultivos celulares), es la proteína no colágena más abundante de la matriz ósea. Es una GLA proteína con tres residuos gammacarboxiglutámicos (en C17, 21 y 24), siendo esta carboxilación dependiente de vitamina K. Este cambio conformacional incrementa su afinidad por el calcio y, al unirse a los cristales de hidroxiapatita, establece puentes entre la matriz mineralizada y el colágeno. Aumenta la formación y el remodelado óseo (es biomarcador de formación ósea), estabiliza el hueso, mejora sus propiedades mecánicas y previene la fractura. Parte de esta osteocalcina, no se carboxila completamente o se decarboxila en el ambiente ácido durante la resorción ósea, pierde afinidad por el hueso y escapa a la circulación para actuar como un factor endocrino. Esta osteocalcina infracarboxilada o no-carboxilada es la forma circulante activa, representa el 40–60 % de la osteocalcina total sanguínea y su medición se considera un indicador de estatus de vitamina K.

**Figura 3:**
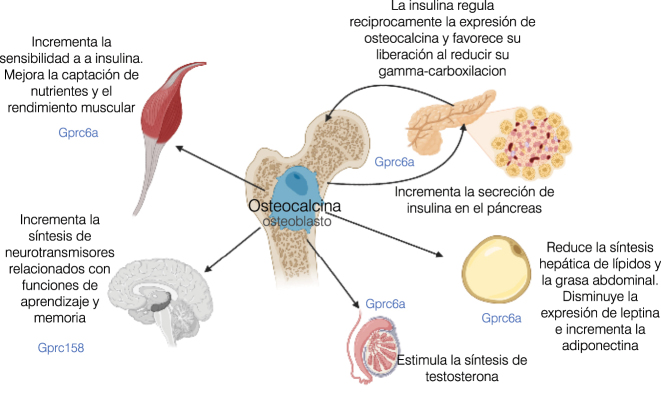
Acciones endocrinas de la osteocalcina. La osteocalcina infra-carboxilada pierde afinidad por el hueso y es liberada a la circulación para ejercer acciones endocrinas, posiblemente a través de los receptores Gprc6a y Gprc158. Participa en la homeostasis energética induciendo la proliferación de células β-pancreática y la secreción de insulina, disminuyendo la síntesis hepática de triglicéridos y la grasa corporal, y reduciendo la expresión de leptina e incrementando la de adiponectina en los adipocitos. Recíprocamente, la insulina regula la expresión osteoblástica de osteocalcina y favorece su liberación al disminuir su carboxilación. La osteocalcina también actúa sobre el músculo esquelético, mejorando su rendimiento. Modelos animales, muestran su acción sobre las células de Leydig aumentando la síntesis de testosterona, así como sobre el sistema nervioso central donde se relaciona con beneficios en las funciones cognitivas. *Diseñada con BioRender.com*.

Este grupo investigador demuestra que los ratones con inactivación de la osteocalcina presentan una menor proliferación de células beta en páncreas, intolerancia a la glucosa y resistencia a la insulina [2]; un fenotipo que se corrige con la administración de osteocalcina. Asociándose a esta intolerancia hidrocarbonada, estos ratones muestran un aumento de grasa visceral, con un incremento en los valores de triglicéridos y leptina y una disminución de adiponectina. Tras estos hallazgos, estudios posteriores demuestran que es necesaria la señalización del receptor osteoblástico de insulina para sintetizar osteocalcina, sugiriendo la existencia de un eje endocrino hueso-páncreas, en el que la insulina regularía recíprocamente la expresión de osteocalcina y favorecería su liberación al promover su infracarboxilación [21]. Junto a estos efectos sobre la proliferación de las células beta-pancreaticas y la secreción de insulina, se han observado otras funciones de la osteocalcina asociadas a la regulación energética, incluyendo efectos sobre el epitelio intestinal (síntesis de incretinas), hepatocitos y adipocitos [22], [23], [24]. La osteocalcina actúa también sobre el músculo esquelético (favoreciendo la captación de nutrientes y su rendimiento), el cerebro (incrementando la síntesis de neurotransmisores relacionados con funciones de aprendizaje y memoria), las neuronas parasimpáticas (regulando la respuesta aguda al estrés) y los testículos (estimulando la producción de testosterona) (Figura 3). Estas acciones se realizan posiblemente a través del Gprc6a, un receptor que se expresa en tejido adiposo, músculo esquelético, y células de Leydig, y que le permitiría actuar a la osteocalcina a modo de sensor de nutrientes, y del Gpr158 en el sistema nervioso central [25, 26]. No obstante, la disminución de osteocalcina circulante en varones tratados con ácido zoledrónico, no se asocia con cambios en los niveles de testosterona, por lo que no parece tener efecto sobre las células de Leydig en el varón adulto [27]. Es más, curiosamente, este fenotipo metabólico característico del modelo murino de Karsenty no se evidencia en otros modelos null de osteocalcina. Estas discrepancias pueden deberse a limitaciones metodológicas (método de ablación, dietas, microbioma…) junto a su complejidad porque la osteocalcina en el ratón está codificada por tres genes (*Bglap, Bglap2* y *Bglap3*); no obstante, el modelo de Karsenty está avalado por casi 2000 estudios posteriores en modelos con ganancia de función, inyecciones de proteína recombinante y ensayos *in vitro* [26]. A diferencia del ratón, la osteocalcina en humanos está codificada por un solo gen (*BGLAP*) y la medición de su forma activa infracarboxilada sigue siendo un reto técnico debido a la poca sensibilidad de los métodos [28]. Aun así, gran número de estudios establecen esta relación de la osteocalcina con el metabolismo energético, especialmente con la diabetes mellitus tipo 2 [29].

Además de la osteocalcina, la lipocalina 2 (LCN2), una proteína ubicua de expresión osteoblástica y asociada con un aumento en la resorción y actividad osteoclástica, se relaciona también con una mayor secreción y sensibilidad a la insulina, tolerancia a la glucosa e incluso control del apetito. De hecho, inicialmente se creyó que esta proteína era exclusiva del tejido adiposo; sin embargo, su expresión es diez veces superior en el osteoblasto que en este tejido o en otros órganos [30].

## Esclerostina: nuevo biomarcador osteocítico

En los últimos años, tras la identificación del FGF23, se está revalorizando el rol del osteocito, cuya gran estructura reticular le permite “sentir” y “responder” liberando diversos factores que actuan a nivel paracrino y endocrino; entre ellos la esclerostina.

### Esclerostina: freno natural del hueso

La esclerostina es una glicoproteína de 22 kd y 213 aminoácidos codificada por el gen *SOST.* Su principal fuente es el osteocito y actúa como un freno natural evitando un crecimiento incontrolado del hueso. Se describió en dos enfermedades raras genéticas, caracterizadas por mutaciones inactivadoras del gen *SOST*: la esclerosteosis [31] y la enfermedad de Van Buchem [32]. Ambas patologías presentan una deficiencia de esclerostina con un marcado aumento de masa ósea, hiperóstosis y estenosis de los foramen, lo que provoca el atrapamiento de los pares craneales con la aparición de sordera y parálisis facial.

La estructura reticular de osteocitos incluida en la matriz ósea constituye un mecanosensor que permite al hueso “sentir” y “responder a la tensión mecánica. Cuando percibe cualquier estímulo mecánico inhibe la expresión de *SOST* y la secreción osteocítica de esclerostina, a través de una señalización en la que podría participar la prostaglandina E2 (PGE2), permitiendo la respuesta osteo-anabólica [33]. Inversamente, la continua ausencia de estímulos mecánicos, como sucede ante la ausencia de gravedad durante los vuelos espaciales, promueve la continua activación del *SOST* y una sobrexpresión de esclerostina, pudiéndose observar pérdidas de masa ósea superiores al 20 % tras una misión espacial [34].

Además de esta respuesta mecanosensora, el osteocito tambien es sensible a estímulos hormonales, coordinando la actividad de remodelado con el mantenimiento de la homeóstasis mineral. El calcitriol induce la expresión osteocítica de esclerostina, aunque existe controversia, dado que el incremento de esclerostina solo se observa en varones y no en mujeres [35]. Es mas, los efectos de la 1,25(OH)_2_D_3_ sobre la expresión de esclerostina difieren entre humanos y ratones, e incluso podrían existir diferencias en el mecanismo de accion entre el calcitriol endógeno (que inhibiría la expresion de esclerostina) y el exógeno y sus análogos (que la promoverían) [33]. Los estrógenos, por el contrario, reducen la expresión de esclerostina, como demuestran diversos estudios en mujeres tratadas con parches transdémicos de estradiol [36], explicando en parte su efecto protector sobre la masa ósea. Este efecto supresor del estradiol sobre el *SOST* está mediado por el receptor β de estrógenos, presentando un efecto opuesto el receptor α [37]. Finalmente, la PTH es un importante inhibidor de *SOST* y de la expresion de esclerostina [38]; de hecho, el efecto anabólico de la PTH administrada de forma intermitente sobre el hueso se fundamenta en esta accion supresora. La infusión de PTH 1–34 produce una disminución significativa de esclerostina a la 6 horas, que se mantiene durante 18 horas [36] y esta rápida disminución de esclerostina tras la administracion de PTH sugiere que, ademas de suprimir la transcripcion del *SOST*, la PTH degradaría la esclerostina osteocítica en el lisosoma [33].

### Acciones paracrinas de la esclerostina

**Figura 4:**
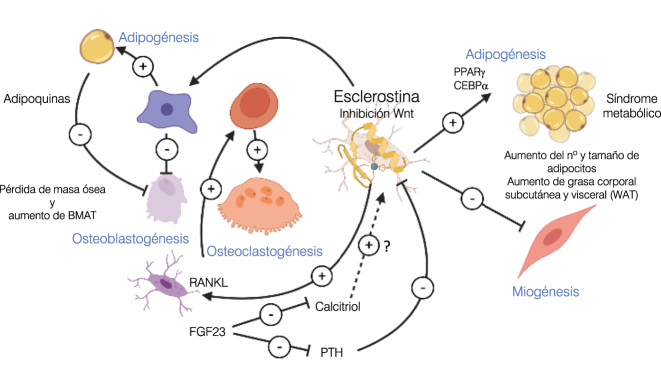
Acciones paracrinas y endocrinas de la esclerostina. A nivel paracrino, el bloqueo de la señalizacion Wnt/β-catenina por la esclerostina inhibe la diferenciación a estirpe osteoblástica de la célula mesangial pluripotencial e induce su diferenciación adipogénica, ocasionando un incremento de BMAT. La secreción de adipoquinas desde este tejido adiposo modula aún más el efecto negativo sobre la osteoblastogénesis y la formación ósea de la esclerostina. En el osteocito, la esclerostina incrementa tanto la expresión osteocítica de RANKL, promoviendo la osteoclastogénesis y la resorción ósea, como la transcripción de FGF23, que a su vez ejerce un efecto supresor sobre la vitamina D y la PTH (que inhibe la expresión de esclerostina). A nivel endocrino, la inhibición del Wnt por la esclerostina induce los master-reguladores de la adipogénesis PPARγ y CEBPα, suprimiendo la diferenciación fibroblástica y la miogénesis, y generando un aumento de grasa corporal, del número y tamaño de los adipocitos y de la incidencia de síndrome metabólico. La percepción de un estímulo mecánico suprime la expresión de esclerostina, iniciándose la respuesta pro-anabólica de la señalización Wnt y anti-catabólica al disminuir la expresión osteocítica de RANKL a nivel óseo. Para cubrir las necesidades bioquímicas (calcio y fósforo) y energéticas que requiere esta respuesta osteo-anabólica, disminuye la síntesis ósea de FGF23 y su efecto fosfatúrico y supresor sobre la PTH y la vitamina D, al tiempo que reduce la adipogénesis y la síntesis *de novo* de ácidos grasos libres. *Diseñado con BioRender.com*. BMAT, Tejido adiposo de la médula ósea; WAT, tejido adiposo blanco.

La esclerostina ejerce una accion antianabólica directa sobre el hueso mediante su unión a la proteina 4 relacionada con el receptor de lipoproteinas de baja densidad (LRP4); de hecho hay casos de esclerosteosis asociados a mutaciones de esta proteína [39]. La LRP4 “secuestra” la esclerostina y facilita su interacción con los correceptores Wnt LRP5/6, bloqueando la unión de los Wnt1 y Wnt3a. Esto promueve la fosforilación de la β-catenina, su degradación en el proteosoma y la supresión de la transcripción de genes pro-osteogénicos y de la osteoblastogénesis (Figura 2B). Junto a esta acción directa inhibidora de la señalización canónica Wnt/β-catenina, la esclerostina promueve indirectamente la osteoclastogénesis y la resorción ósea al inducir la expresión y secreción osteocítica de RANKL [40] (Figura 4).

La inhibición del Wnt en el tejido óseo promueve la diferenciación de la célula pluripotencial hacia estirpes no osteogénicas, induciendo, entre otras, la adipogénesis. El depósito del tejido adiposo de la médula ósea (BMAT) despierta últimamente gran interés. Su localización anatómica es perfecta para establecer un eje “grasa-hueso”, en el que el adipocito modularía el remodelado óseo mediante la secreción de adipoquinas y citoquinas. La BMAT, que representa el 50–70 % de la médula ósea en el adulto sano, aumenta con la edad y se asocia inversamente con la masa ósea, incrementándose en situaciones de baja masa ósea, como la osteoporosis o la anorexia nerviosa [41]. De hecho, se ha demostrado que la esclerostina promueve la diferenciación a adipocito en líneas celulares de pre-adipocitos, justificando la disminución de BMAT que presentan las ratas null para esclerostina [42]. Asimismo, la administración de anticuerpos anti-esclerostina reduce los depósitos de BMAT, el número y tamaño de los adipocitos. Análogamente, en humanos se ha observado una asociación directa entre el incremento de esclerostina y la BMAT en pacientes con ERC, lo que sugiere el posible papel regulador de la esclerostina sobre la BMAT [41].

### Acciones endocrinas de la esclerostina sobre el metabolismo mineral (Figura 4)

El ratón *Sost* ^*−/−*^ representa un modelo ideal para estudiar la interacción de la supresión de esclerostina con el balance mineral e ilustrar las posibles acciones endocrinas de la esclerostina. La elevada tasa de aposición ósea de estos ratones, requiere un balance positivo de calcio y fósforo. Así, este modelo transgénico muestra un incremento neto en los valores de calcitriol debido al aumento de la expresión renal de 1alfa-hidroxilasa renal y la reducción en la expresión de 24-hidroxilasa. Asimismo, se observa un aumento en la reabsorción tubular de calcio, aunque los niveles circulantes de calcio no están aumentados (seguramente por secuestro óseo) y la PTH se sitúa dentro de su intervalo de referencia [43, 44]. Junto a estas acciones calciotrópicas, se observa una disminución en los niveles circulantes de FGF23, evitando así un posible balance negativo del fósforo generado por su efecto fosfatúrico e inhibidor sobre la vitamina D.

El problema es que los estudios sobre homeóstasis mineral en modelos animales con sobrexpresión de esclerostina son escasos, limitando el conocimiento del papel endocrino de la esclerostina sobre la misma. No obstante, en un modelo de línea celular de osteocito (IDG-SW3) (que expresa FGF23 a nivel mRNA y proteína), se ha demostrado que la esclerostina induce la síntesis ósea de FGF23 suprimiendo la expresión de reguladores negativos de su transcripción como el PHEX o el DMP1 y simultáneamente inhibe su degradación proteolítica, al inducir la expresión de GALNT3 y la estabilización de la molécula [45].

### Acciones endocrinas de la esclerostina sobre el metabolismo energético (Figura 4)

La señalización Wnt suprime la inducción de los master-reguladores de la adipogénesis (PPARγ y CEBPα), promoviendo la diferenciación fibroblástica y la miogénesis. El efecto inhibidor de la esclerostina sobre la Wnt/β-catenina también afecta al tejido adiposo blanco (WAT), tanto subcutáneo como visceral, y al metabolismo energético. Los ratones *Sost* ^−/−^ tienen menos masa grasa corporal y, menor número y tamaño de adipocitos en el panículo adiposo gonadal, inguinal y retroperitoneal. Además, generan resistencia a las dietas ricas en grasa, con una reducción de ácidos grasos libres y glucosa circulantes, asociada a una mayor oxidación y una menor síntesis *de novo* de los ácidos grasos. A la inversa, la sobrexpresión de esclerostina condiciona un incremento de grasa corporal, con aumento del número y tamaño de los adipocitos en el tejido adiposo blanco [3]. Esta acción pro-anabólica y anti-catabólica de la esclerostina sobre el adipocito es un efecto directo, ya que la ablación del LRP4 en tejido adiposo reduce la hipertrofia del adipocito *in vitro* e *in vivo*, mejora el metabolismo lipídico y aumenta la sensibilidad a la insulina. Es más, la inhibición selectiva del LRP4 a nivel del osteoblasto condiciona una sobrexpresión de esclerostina circulante y el efecto metabólico opuesto [46].

Esta actividad adipogénica de la esclerostina asociada a la inhibición Wnt, también se ha demostrado en humanos. Los portadores de variantes génicas con ganancia del LRP5 y fenotipo de masa ósea aumentada, muestran un menor depósito de grasa corporal. Por el contrario, en las variantes con pérdida de función LRP5 y baja masa densidad mineral ósea, se observa un aumento de grasa abdominal [47]. Curiosamente, el LRP5 se expresa más en el WAT subcutáneo que en el visceral, condicionando la distribución de la grasa corporal; es más, en biopsias de voluntarios sanos se demuestra mayor expresión de LRP5 en la WAT subcutánea abdominal que en la glúteo-femoral, afectando al riesgo metabólico (por un perfil menos favorable de adipoquinas de la grasa abdominal). Los estudios clínicos demuestran una correlación positiva de la esclerostina con la masa grasa y la incidencia de síndrome metabólico. Los pacientes con diabetes tipo 2 muestran valores significativamente superiores de esclerostina circulante frente a controles no diabéticos [48, 49]. Sin embargo, los resultados de estos estudios deben interpretarse cuidadosamente, por errores en la exactitud y consistencia de los métodos de medición de esclerostina; de hecho, existen diferencias significativas en los resultados dependiendo del método [50] y ni siquiera conocemos hasta qué punto la esclerostina circulante representa la proteína activa.

### Esclerostina como biomarcador y diana terapéutica

Los niveles circulantes de esclerostina son superiores, a cualquier edad, en varones frente a mujeres, representando posiblemente su mayor masa ósea, ya que la principal fuente de esclerostina es el pool de osteocitos. Eso explicaría que, aunque lo esperable sea que los niveles elevados de esclerostina circulante se relacionen con baja masa ósea, la mayoría de estudios demuestren una asociación positiva con la densidad mineral ósea [51]. La esclerostina se encuentra más elevada en mujeres postmenopáusicas que en premenopáusicas, y se incrementa con la edad, posiblemente por la contribución de tejidos extraóseos como la calcificación vascular, porque los niveles óseos de mRNA de esclerostina son similares a cualquier edad [52].

En la ERC los niveles circulantes de esclerostina se incrementan conforme disminuye el filtrado glomerular, alcanzando hasta 4 veces su valor en prediálisis [53, 54]. Este incremento no se justifica por la pérdida de aclaramiento ya que, por su tamaño molecular, puede filtrarse y reabsorberse; es más, un estudio demuestra que la excreción renal de esclerostina aumenta conforme declina la función renal [55]. Diversos estudios sugieren un aumento inicial en la síntesis osteocítica de esclerostina en el transcurso de la ERC, aunque posteriormente, el incremento de la PTH inhibe *SOST* y la expresión de esclerostina [54, 56]; de hecho, la esclerostina correlaciona negativamente con la PTH y los marcadores de remodelado óseo. Otra posibilidad es un origen extraóseo, dado que en el proceso de calcificación vascular, propio de las alteraciones del metabolismo mineral en la ERC, la célula de músculo liso vascular sufre una transformación fenotípica a osteoblasto-like y, por tanto, podría expresar esclerostina. Es más, la inhibición de la señalización Wnt/β-catenina a nivel vascular podría representar un mecanismo de defensa en el que la esclerostina ejercería un papel protector sobre la calcificación [57, 58].

La esclerostina también se eleva en otras alteraciones óseas, como la enfermedad de Paget o en las metástasis óseas líticas. En el mieloma múltiple aumenta la apoptosis de los osteocitos, ocasionando una sobrexpresión de RANKL y de esclerostina. En estos pacientes los niveles aumentados de esclerostina se asocian a peor supervivencia [59]. Finalmente, se ha sugerido una asociación entre la esclerostina y alteraciones neurodegenerativas, como la enfermedad de Alzheimer, basándose en posibles acciones pleiotrópicas de la señalización Wnt/β-catenina sobre la plasticidad sináptica y la memoria, aunque esto está por demostrar [60].

Por todo ello, la esclerostina emerge como un potencial biomarcador para futuro uso clínico, reflejando los eventos óseos patológicos y su interacción con otros tejidos del organismo, como avalan estudios recientes [61, 62]. Sin embargo, la falta de estandarización de los diversos métodos que miden la esclerostina limita su utilidad clínica [51]. Los diferentes métodos existentes presentan importantes discrepancias en exactitud y especificidad, lo que influye en su interpretación y justifican los resultados contradictorios en algunos estudios. Solamente tres de ellos miden la forma intacta de esclerostina: dos ELISAs (Biomédica y TECO medical) y el único método automatizado (Diasorin) y ninguno de ellos está validado para uso clínico. La armonización de estos métodos en el futuro es crucial.

En paralelo a su importancia como biomarcador, actualmente se ha desarrollado un anticuerpo anti-esclerostina, como diana terapéutica: Romosozumab (AMG785), una IgG2 monoclonal humanizada que se une y neutraliza la acción de la esclerostina humana, aprobada en 2020 para Europa y recientemente comercializada en España. Este anticuerpo ejerce un mecanismo dual sobre las células óseas: pro-anabólico y anti-resortivo, mostrando una recuperación rápida de la densidad mineral ósea y una reducción significativa de la fractura. Una revisión reciente de la eficacia y seguridad de su utilización en el manejo de la osteoporosis revela incrementos en la densidad mineral ósea de entre 13,1 y 13,3 % a nivel lumbar y de 2,2–5,9 % y 2,5–6,9 % en cuello femoral y cadera total, respectivamente, junto con una reducción de fracturas vertebrales, principales y de cadera [63]. Además de ser un fármaco muy prometedor en el manejo de la osteoporosis, el Romosozumab también parece mostrar un efecto positivo sobre la afectación ósea del mieloma múltiple, donde los valores aumentados de esclerostina contribuirían al desarrollo de la lesión lítica.

## Conclusiones

El cambio de paradigma en la homeostasis mineral también alcanza al hueso. Se revaloriza el papel del osteocito como célula endocrina que controla el remodelado óseo y el balance del fósforo, sintetizando moléculas clave (DKK1, esclerostina, FGF23), y se establece su relación con la BMAT, tejido que empieza a cobrar relevancia. Mas allá de sus acciones paracrinas, diversas proteínas osteocíticas y osteoblásticas participan en la homeóstasis energética controlando el aporte de nutrientes, su almacenamiento, la secreción de insulina y los depósitos de grasa, y regulan su consumo en paralelo a la actividad de remodelado. A través de estas “osteoquinas” el hueso interactúa con otros órganos como el páncreas y el hígado, o el músculo esquelético, modulando su gasto energético y conectando la actividad física con la salud ósea. E incluso con el cerebro, relacionándose con el control del apetito y las funciones cognitivas.

Aunque diversos estudios clínicos confirman el papel de estas osteoquinas en humanos, se precisan datos adicionales que los validen, ya que gran parte del conocimiento se fundamenta en modelos animales. Existen limitaciones técnicas ya que los métodos que determinan osteocalcina infracarboxilada en humanos son poco sensibles y los de esclerostina muestran resultados discrepantes por diferencias en su estandarización y especificidad. El desarrollo de métodos fiables y su armonización contribuirá a definir el papel de estas proteínas y su utilidad como biomarcadores óseos en el manejo clínico de diversas patologías, poniendo en valor el papel del laboratorio clínico.

Junto a su valor como biomarcadores, su conocimiento plantea hipótesis terapéuticas, como el posible beneficio de la administración exógena de osteocalcina sobre la secreción de insulina o la sarcopenia. De hecho, el FGF23 y la esclerostina ya son dianas terapéuticas tras el desarrollo de anticuerpos contra estas proteínas en el tratamiento del XLH y la osteoporosis respectivamente. Esto es solo el principio del diálogo del hueso con otros tejidos.
